# Serum Adiponectin Level and Clinical, Metabolic, and Hormonal Markers in Patients with Polycystic Ovary Syndrome

**Published:** 2013-12-22

**Authors:** Yunus Yildiz, Gülnur Ozaksit, Bekir Serdar Unlu, Emre Ozgu, Hasan Energin, Metin Kaba, Mustafa Ugur

**Affiliations:** Department of Obstetrics and Gynecology, Zekai Tahir Burak Women’s Health Education and Research Hospital, Ankara, Turkey

**Keywords:** Adiponectin, Insulin Resistance, Polycystic Ovary Syndrome

## Abstract

**Background:**

To investigate the relationship between adiponectin, metabolic and hor- monal parameters, and insulin resistance in patients with non-treated polycystic ovary syndrome.

**Materials and Methods:**

In this cross-sectional observational study, 81 patients admitted to out-patient clinic with complaints of menstrual irregularity, hirsutism and obesity were enrolled. Serum adiponectin, biochemical and hormonal parameters, and 75 gram oral glu- cose tolerance test (OGTT) were measured. Spearman’s correlation coefficient was used for statistical analysis.

**Results:**

We observed inverse correlations between serum adiponectin level and body mass index, homeostasis model assessment insulin-resistance score, insulin level, fast- ing glucose level, and prolactin level (p=0.001, p=0.02, p=0.04, p=0.02, and p=0.005, respectively). No significant correlations were found between serum adiponectin level and age, height, weight, Ferriman-Gallwey score, 2 hours OGTT test value and free tes- tosterone level (p=0.3, p=0.6, p=0.2, p=0.8, p=0.9, and p=0.01, respectively).

**Conclusion:**

The present study demonstrated that in polycystic ovary syndrome patients, when serum adiponectin level decreased, degree of insulin resistance increased. Our find- ings indicate that serum adiponectin level is likely to be an adequate marker for deter- mination of the degree of insulin resistance, and may be a predictor of diseases, such as type 2 diabetes mellitus (T2DM) and metabolic syndrome, which develop on the basis of insulin resistance.

## Introduction

Polycystic Ovary Syndrome (PCOS), as a heterogeneous disease, is characterized by menstrual dysfunction, clinical or metabolic hyperandrogenism, and in some patients, and sometimes polycystic appearence in any or both ovaries in radiological imaging (1, 2). Insulin resistance and hyperinsulinemia are the most common characteristic findings in patients with PCOS (3). In recent studies, they have reported an increased insulin response against serum glucose level and an insulin resistance in 26-60% of obese and non-obese patients with PCOS (4). However, the degree of insulin resistance has been shown to be higher in obese PCOS patients than in non-obese PCOS patients (5).

Adiponectin is one of adipocytokines secreted from visceral adipose tissue, and is only secreted by mature adipocytes. The serum concentration of adiponectin decreases in obese patients, opposite to other adipocytokines, even though, it is only secreted by adipose tissue. Adipose tissue takes place as the central organ of systemic insulin sensitivity by synthesis and secretion of adiponectin (6).

There are many studies on adiponectin in patients with PCOS. However, little is known about the biology of adiponectin and its role in further systemic metabolic problems (2).

Adiponectin was correlated with insulin sensitivity (7) and the low levels of adiponectin are associated with increased risk of type 2 diabetes (8).

In recent studies, serum adiponectin level progressively decreased in patients according to the severity of obesity, insulin resistance, diabetes mellitus and cardiovascular diseases. Reduced serum level of adiponectin was determined before onset of symptoms and the beginning of clinical findings. Therefore, low serum adiponectin level may be a predictive factor for type 2 diabetes mellitus (T2DM) and other cardiovascular diseases (9).

In our study, we have examinated the degree of insulin resistance in patients with PCOS. Our most important aim was to evaluate the role of insulin resistance in PCOS patients and to determine the predictive capacity of adiponectin for further metabolic abnormalities.

## Materials and Methods

### Subject and study design

This cross-sectional observational study was performed with a total of 81 patients. The participants were selected via simple random sampling method among total of five hundred PCOS patients who were admitted to Adolescent Outpatient Clinics of the Zekai Tahir Burak Women’s Health Education and Research Hospital, Ankara, Turkey, with menstrual irregularity, hirsutism and obesity between August 2010 and August 2011. Ethics approval was obtained from our institutional review board. Informed written consent was obtained from all participants. The participants were selected according to the 2006 Androgen Excess Society (AES) criteria for PCOS patients, so inclusion criteria were as follows: i. no PCOS treatment for the last 3 months, ii. normal thyroid function, iii. normal prolactin serum levels, iv. the absence of other diseases causing ovulatory disfunction, and v. abnormal androgen metabolism. Demographic features of patients, like weight and height, as well as clinical criteria, including hirsutism (Ferriman-Gallway >8), acne or male pattern alopecia, were recorded. The following tests were performed for all participants who were instructed to follow a 12 hours fasting and a 24 hours avoidance of excessive exercise and alcohol: i. biochemical measurements including serum fasting insulin levels and fasting glucose levels using chemiluminescence detection method, ii. hormonal measurements including free testosterone using radioimmunassay method, serum prolactin levels (PRL), and 17-hydroxy progesterone (17-OH PROG), and iii. 75-gram oral glucose tolerance test (OGTT). Many methods exist for the measurement the degree of insulin resistance. Although the best method is the euglysemic clamping test, due to technical difficulties, we preferred to use the homeostasis model assessment insulin-resistance (HOMA-IR) score, fasting plasma glucose level, fasting plasma insulin level and 24 hours OGTT test value. Height was measured by stadiometer and weight was measured in our outpatient clinic. Body mass index (BMI) was calculated using the following formula: weight (kg)/height (meter) squared. Plasma adiponectin levels were measured by enzyme linked immunoabsorbent assay (ELISA) kit (Millipore, USA) according to the manufacturer’s instruction.

### Data preparation and statistical analysis

Data analysis was conducted by Statistical Package for the Social Sciences (SPSS; version 19.0) software. Spearman’s product-moment correlation coefficient was used to explore the relationship between variables. Within a 95% confidence interval, correlation between two groups was accepted as significant if the p value was <0.05.

## Results

The prospective study included 81 patients with PCOS. Anthropometric, hormonal and biochemical parameters and serum adiponectin value of patients with PCOS are shown in table 1.

Relationship between serum adiponectin level with clinic, metabolic and biochemical parameters are shown in table 2.

Significant reverse correlation was determined between the following variables: i. serum adiponectin and fasting insulin levels (p=0.04), ii. serum adiponectin and fasting glucose levels (p=0.02), and iii. serum adiponectin levels and HOMA-IR score (p=0.02).

Significant reverse correlation was observed between adiponectin level and degree of insulin resistance in patients with PCOS. There is a significant reverse correlation between serum adiponectin and BMI for patients with PCOS (p=0.001). Significant reverse correlation was observed between adiponectin level and prolactin level (p=0.005), but this situation was not an essential objective of the study; therefore, further studies should be performed to investigate any possible relation between adiponectin and prolactin levels for predicting possible metabolic abnormalities.

**Table 1:** Characteristics of PCOS patients and statistical analysis of other parameters examined in the study


	Count	Median	Minimum	Maximum	Range

**Age (Y)**	81	19	13	24	11
**Height (m)**	81	1.61	1.45	1.81	0.36
**Weight (kg)**	81	60	45	96	51
**BMI (kg/m^2^)**	81	27.2	23.4	31.0	21
**Free testosterone (pg/ml)**	81	2.00	.33	10.14	9.81
**Ferriman-Gallaway**	81	6	2	10	8
**Fasting glucose (mg/dl)**	81	88.80	72.90	135.00	62.10
**Insulin (uIU/ml)**	81	9.69	2.00	109.37	107.37
**OGTT 2. Hour (mg/dl)**	81	100.85	52.30	196.90	144.60
**Prolactin (ng/ml)**	81	11,29	4,88	58,10	53.22
**17-OH Progesterone (ng/ml)**	81	2,00	0,5	6.47	5.97
**HOMA-IR [(uIU/ml x mg/dl)/22,5]**	81	5.54	5.54	5.54	31.46
**Adiponectin (ng/ml)**	81	20.90	12.40	40.40	28


**Table 2:** Relationship between serum adiponectin level and clinic, metabolic, and biochemical parameters


Parameters	Rho	P value

**Age (Y)**	0.127	0.3
**Height (m)**	-0.05	0.67
**Weight (kg) **	-0.137	0.27
**BMI (kg/m^2^)**	-0.354	0.001^*^
**Ferriman-Gallaway **	-0.017	0.88
**Prolactin (ng/ml)**	-0.339	0.005^*^
**Insulin (uIU/ml)**	-0.228	0.04^*^
**Fasting plasma glucose **	-0.25	0.02^*^
**HOMA-IR [(uIU/ml x mg/dl)/22,5]**	-0.264	0.02^*^
**OGTT 2. Hour (mg/dl)**	0.001	0.99
**Free testosterone (pg/ml)**	0.173	0.17


## Discussion

The present study demonstrated that in polycystic ovary syndrome patients, when serum adiponectin level decreased, degree of insulin resistance increased, and that there was also a significant reverse correlation between serum adiponectin and BMI in patients with PCOS.

PCOS includes multiple metabolic abnormalities in addition to polycystic ovaries, so the most important indications in recent studies are insulin resistance and hyperinsulinemia (10, 11). In our study, degree of insulin resistance and pattern were demonstrated in PCOS patients. The correlations between metabolic parameters and serum adiponectin were also investigated, and the result confirms that metabolic parameters are significantly correlated with adiponectin serum level.

The group of mediators synthesized and secreted from adipose tissue are leptin, adiponectin, tumor necrosis factor alpha (TNF-α), ghrelin, retinol-binding protein-4 (RBP-4), resistin, visfatin, and apelin. These molecules are involved in many physiological processes such as lipid metabolism atherosclerosis, blood pressure regulation, insulin sensitivity, and angiogenesis and affect immunity and inflammation.

Adiponectin serum level plays an important role in the development of T2DM due to its unique contribution to increasing insulin sensitivity and to improving islet beta cell dysfunction and fatty acid beta-oxidation (12-14). It is well established that lowered adiponectin concentration is associated with T2DM, obesity, dyslipidemia , insulin resistance and cardiovascular diseases (15-20).

Panidis et al. (21) demonstrated that PCOS patients with higher insulin resistance calculated by HOMA-IR score have lower serum adiponectin levels than the normal population. In our study, we observed similar findings, and also determined a significant correlation between HOMA-IR score and adiponectin serum level.

Xita at al. (22) demostrated that obese PCOS patients have lower adiponectin serum level than normal weight PCOS patients. In researches (2, 5, 9) has showed that there is a general correlation between insulin resistance and adiponectin level. We observed the same characteristics in our study.

Free testosterone is the best marker in the definition of hirsutism. In a recent study, no significant relation between serum adiponectin and androgen level was identified in healthy women (23), whereas higher androgen serum level was found with increasing adiponectin serum level in PCOS patients (24, 25); however, in some studies, no relationship was demonstrated between these two parameters (26, 27). According to Nishizawa et al. (28) serum adiponectin level can be reduced by androgen therapy; in addition, there is evidence that supplementation of flutamide and metformine plus oral contraceptive therapy reduce adiponectin serum level in adolescents with hirsutism (29). In our study, no significant correlation was observed between serum adiponectin and free testosterone levels.

Since we did not encounter any study about correlation between adiponectin and prolactin in literature research, we tried to investigate any correlation between these serum markers in PCOS patients. de Assunção Alves Rodrigues LF et al. (30) have showed that prolactinoma is associated with hypoadiponectinemia. In this study, adiponectin level was compared between patients with prolactinoma and controls, and the result demonstrated that patients with prolactinomas showed higher insulin level and HOMAIR score with lower adiponectin level (29). Although the role of prolactin on glucose methabolism is still unclear, it has been demonstrated high prolactin level improves β-cell mass by increasing β-cell proliferation and exacerbates insulin resistance (30). But, in our study, patients whose serum prolactin levels were outside the normal range were excluded. Serum adiponectin level was determined to have significantly reverse correlation with prolactin level within the normal range. This finding shows prolactine has a role in insulin resistance. Therefore, we consider serum level of prolactin as directive marker for metabolic abnormalities in PCOS patient.

The patients with heterogenous distribution of BMI may be an acceptable argumentative term of our study. Although all PCOS patients were considered according to current diagnosis criteria, patients with standardized BMI value were unavailable in our study.

## Conclusion

Although the exact pathogenesis of PCOS is obscure, it is generally accepted that insulin resistance, hyperinsulinemia and clinical and/or biochemical hyperandrogenism may play a role in its pathogenesis. The most implicated factor in our study was insulin resistance, which is consistent with the mentioned-literatures. There was also a correlation between adiponectin level and the degree of insulin resistance. An investigation of the degree of insulin resistance may help physicians to predict further systemic morbidity of PCOS. We consider serum adiponectin level as an adequate serum marker for demonstrating the degree and pattern of insulin resistance in PCOS patients.

## References

[1] Revelli A, Dolfin E, Gennarelli G, Lantieri T, Massobrio M, Holte JG (2008). Low-dose acetylsalicylic acid plus prednisolone as an adjuvant treatment in IVF: a prospective, randomized study. Fertil Steril.

[2] Salehpour S, Broujeni PT, Samani EN (2008). Leptin, ghrelin, adiponectin, homocysteine and insulin resistance related to polycystic ovary syndrome. Int J Fertil Steril.

[3] Bo S, Menato G, Botto C, Cotrino I, Bardelli C, Gambino R (2006). Mild gestational hyperglycemia and the metabolic syndrome in later life. Metab Syndr Relat Disord.

[4] Beamer BA, Yen CJ, Andersen RE, Muller D, Elahi D, Cheskin LJ (1998). Association of the Pro12Ala variant in the peroxisome proliferator-activated receptor-gamma2 gene with obesity in two Caucasian populations. Diabetes.

[5] De Leo V, la Marca A, Petraglia F (2003). Insulin-lowering agents in the management of polycystic ovary syndrome. Endocr Rev.

[6] Arita Y, Kihara S, Ouchi N, Takahashi M, Maeda K, Miyagawa J (1999). Paradoxical decrease of an adipose-specific protein, adiponectin, in obesity. Biochem Biophys Res Commun.

[7] Pajvani UB, Hawkins M, Combs TP, Rajala MW, Doebber T, Berger JP (2004). Complex distribution, not absolute amount of adiponectin, correlates with thiazolidinedionemediated improvement in insulin sensitivity. J Biol Chem.

[8] Nakashima R, Kamei N, Yamane K, Nakanishi S, Nakashima A, Kohno N (2006). Decreased total and high molecular weight adiponectin are independent risk factors for the development of type 2 diabetes in Japanese-Americans. J Clin Endocrinol Metab.

[9] Yamauchi T, Kamon J, Ito Y, Tsuchida A, Yokomizo T, Kita S (2003). Cloning of adiponectin receptors that mediate antidiabetic metabolic effects. Nature.

[10] Johnstone EB, Davis G, Zane LT, Cedars MI, Huddleston HG (2012). Age-related differences in the reproductive and metabolic implications of polycystic ovarian syndrome: findings in an obese, United States population. Gynecol Endocrinol.

[11] Li W, Ma L, Li Q (2012). Insulin resistance but not impaired β-cell function: a key feature in Chinese normal-weight PCOS women with normal glucose regulation. Gynecol Endocrinol.

[12] Abbasi F, Chu JW, Lamendola C, McLaughlin T, Hayden J, Reaven GM (2004). Discrimination between obesity and insulin resistance in the relationship with adiponectin. Diabetes.

[13] Tschritter O, Fritsche A, Thamer C, Haap M, Shirkavand F, Rahe S (2003). Plasma adiponectin concentrations predict insulin sensitivity of both glucose and lipid metabolism. Diabetes.

[14] Weyer C, Funahashi T, Tanaka S, Hotta K, Matsuzawa Y, Pratley RE (2001). Hypoadiponectinemia in obesity and type 2 diabetes: close association with insulin resistance and hyperinsulinemia. J Clin Endocrinol Metab.

[15] Li S, Shin HJ, Ding EL, van Dam RM (2009). Adiponectin levels and risk of type 2 diabetes: a systematic review and metaanalysis. JAMA.

[16] Lu JY, Chang LC, Huang YS, Chi YC, Su TC (2008). Adiponectin: a biomarker of obesity-induced insulin resistance in adipose tissue and beyond. J Biomed Sci.

[17] Yang WS, Lee WJ, Funahashi T, Tanaka S, Matsuzawa Y, Chao CL (2002). Plasma adiponectin levels in overweight and obese Asians. Obes Res.

[18] Matsubara M, Maruoka S, Katayose S (2002). Decreased plasma adiponectin concentrations in women with dyslipidemia. J Clin Endocrinol Metab.

[19] Hotta K, Funahashi T, Arita Y, Takahashi M, Matsuda M, Okamoto Y (2000). et al.Plasma concentrations of a novel, adipose- specific protein, adiponectin, in type 2 diabetic patients. Arterioscler Thromb Vasc Biol.

[20] Kumada M, Kihara S, Sumitsuji S, Kawamoto T, Matsumoto S, Ouchi N (2003). Association of hypoadiponectinemia with coronary artery disease in men. Arterioscler Thromb Vasc Biol.

[21] Panidis D, Kourtis A, Kukuvitis A, Farmakiotis D, Xita N, Georgiou I (2004). Association of the T45G polymorphism in exon 2 of the adiponectin gene with polycystic ovary syndrome: role of delta4-androstenedione. Hum Reprod.

[22] Xita N, Georgiou I, Chatzikyriakidou A, Vounatsou M, Papassotiriou GP, Papassotiriou I (2005). Effect of adiponectin gene polymorphisms on circulating adiponectin and insulin resistance indexes in women with polycystic ovary syndrome. Clin Chem.

[23] Gavrila A, Chan JL, Yiannakouris N, Kontogianni M, Miller LC, Orlova C (2003). Serum adiponectin levels are inversely associated with overall and central fat distribution but are not directly regulated by acute fasting or leptin administration in humans: cross-sectional and interventional studies. J Clin Endocrinol Metab.

[24] Glintborg D, Andersen M, Hagen C, Frystyk J, Hulstrøm V, Flyvbjerg A (2006). Evaluation of metabolic risk markers in polycystic ovary syndrome (PCOS).Adiponectin, ghrelin, leptin and body composition in hirsute PCOS patients and controls. Eur J Endocrinol.

[25] Ardawi MS, Rouzi AA (2005). Plasma adiponectin and insulin resistance in women with polycystic ovary syndrome. Fertil Steril.

[26] Scabini S, Rimini E, Massobrio A, Romairone E, Linari C, Scordamaglia R (2011). Primary omental torsion: a case report. World J Gastrointest Surg.

[27] Serventi A, Rassu PC, Giaminardi E, Massobrio A, Vitali GC, Stabilini L (2011). Haemorrhoidal disease: role of conservative outpatient treatments. Ann Ital Chir.

[28] Nishizawa H, Shimomura I, Kishida K, Maeda N, Kuriyama H, Nagaretani H (2002). Androgens decrease plasma adiponectin, an insulin-sensitizing adipocyte-derived protein. Diabetes.

[29] Ibáñez L, De Zegher F (2004). Flutamide-metformin plus an oral contraceptive (OC) for young women with polycystic ovary syndrome: switch from third- to fourth-generation OC reduces body adiposity. Hum Reprod.

[30] de Assunção Alves Rodrigues LF, Campos SM, Bizzi MF, Sales do Amaral PH, Giannetti AV (2012). Prolactinoma: a condition associated with hypoadiponectinemia. Horm Metab Res.

[31] Park S, Kim da S, Daily JW, Kim SH (2011). Serum prolactin concentrations determine whether they improve or impair beta-cell function and insulin sensitivity in diabetic rats. Diabetes Metab Res Rev.

